# Influence of Uric Acid on Vascular and Cognitive Functions: Evidence for an Ambivalent Relationship

**DOI:** 10.3390/metabo14110642

**Published:** 2024-11-20

**Authors:** Francesco Baratta, Federica Moscucci, Evaristo Ettorre, Raffaella Bocale, Arrigo F. G. Cicero, Pietro Cirillo, Federica Fogacci, Ilaria Lospinuso, Carmine Savoia, Alessandro Mengozzi, Agostino Virdis, Claudio Borghi, Giovambattista Desideri

**Affiliations:** 1 Geriatric Unit, Department of Internal Medicine and Medical Specialties, AOU Policlinico Umberto I, 00161 Rome, Italy; francesco.baratta@uniroma1.it (F.B.); federica.moscucci@uniroma1.it (F.M.); evaristo.ettorre@uniroma1.it (E.E.);; 2 Department of Clinical, Internal Medicine, Anesthesiologic and Cardiovascular Sciences, Sapienza University of Rome, 00161 Rome, Italy; 3 Unit of Endocrinology, Agostino Gemelli University Hospital Foundation, Scientific Institute for Research, Hospitalization and Healthcare (IRCCS), Catholic University of the Sacred Heart, 00168 Rome, Italy; raffaella.bocale@policlinicogemelli.it; 4 Cardiovascular Medicine Unit, IRCCS AOU BO, 40138 Bologna, Italy; arrigo.cicero@unibo.it (A.F.G.C.); claudio.borghi@unibo.it (C.B.); 5 Hypertension and Cardiovascular Risk Factor Research Center, Medical and Surgical Sciences Department, Alma Mater Studiorum, University of Bologna, 40138 Bologna, Italy; federica.fogacci@studio.unibo.it; 6 Nephrology, Dialysis and Transplantation Unit, Department of Emergency and Organ Transplantation, “Aldo Moro” University of Bari, 70122 Bari, Italy; pietro.cirillo@policlinico.ba.it; 7 Clinical and Molecular Medicine Department, Faculty of Medicine and Psychology, Sant’Andrea Hospital, Sapienza University of Rome, 00189 Rome, Italy; carmine.savoia@uniroma1.it; 8 Department of Clinical and Experimental Medicine, University of Pisa, 56126 Pisa, Italy; alessandro.mengozzi@unipi.it (A.M.); agostino.virdis@unipi.it (A.V.)

**Keywords:** uric acid, cardiovascular disease, cerebrovascular disease, cognitive function

## Abstract

The growing recognition of the public health impact of cognitive impairment and dementia has sparked a global initiative to identify risk factors and develop strategies to prevent or slow the progression of these cognitive disorders. Uric acid, the end product of the metabolism of purine nucleotides, has been reported as a key factor of many conditions potentially involved in cognitive dysfunction/dementia. In addition, some studies support the hypothesis that elevated uric acid levels could reduce the risk of Alzheimer’s disease, slow down the decline of cognition, and delay the progression of Alzheimer’s disease, while other evidence achieves opposite positions. These discrepancies might reflect a biological ambivalence for uric acid depending on a very complex interplay of factors that include its concentrations achieved in biological fluids, the nature, and concentration of free radicals, the presence and concentration of other antioxidant molecules, potentially responsible for bi-directional effects of uric acid on brain health/functioning. In this narrative review, we attempt to elucidate the influential role of uric acid metabolism in cognitive functioning by discussing pathophysiological mechanisms putatively involved, being well aware that none of them can be considered one-sided due to the complexity of the human organism.

## 1. Introduction

Dementia, characterized by progressive cognitive decline and functional impairment, represents a substantial public health challenge with limited curative options. Due to rapid global demographic changes, the number of people with dementia is expected to double every 20 years, making dementia one of the largest socioeconomic and health challenges worldwide. Nevertheless, it has been reported that age-standardized rates of dementia have decreased in several high-income countries over the past 20 years [1], probably related, at least in part, to earlier detection and treatment of illness risk factors [2]. With the growing elderly population, there is a need for evidence-based methods to slow or prevent cognitive decline in its early stages [3]. In this regard, a consistent bulk of data support the connection between cognition and cardiovascular risk factors [4]. In particular, high blood pressure has been associated with the pathophysiology of Mild Cognitive Impairment, Alzheimer’s disease, and vascular dementia [4,5]. This connection may be influenced by endothelial dysfunction and microvascular diseases, resulting in reduced vascular reserve [4,5].

**Figure 1:**
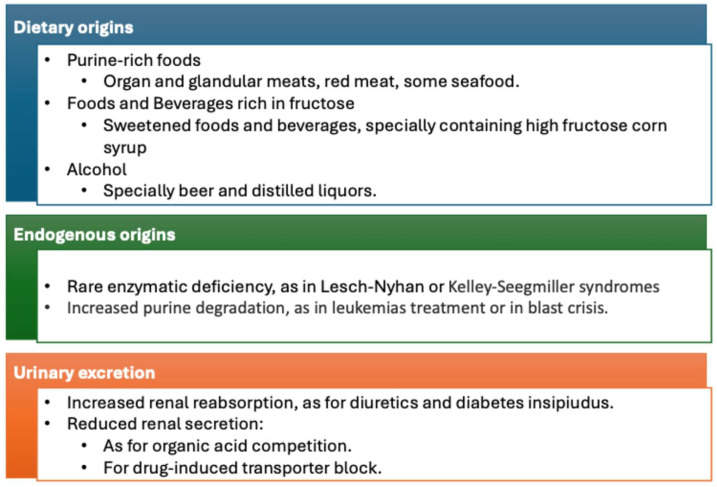
Mechanism leading to hyperuricemia.

There is growing evidence that hyperuricemia may play a significant role in the development and progression of cardiorenal metabolic syndrome [6,7]. High uric acid levels are linked to inflammation, oxidative stress, insulin resistance, dysglycemia, endothelial dysfunction, vascular stiffness, cardiac diastolic dysfunction, renal hyperfiltration, and proteinuria, all components of the cardiorenal syndrome [6,7,8,9,10] and metabolic pathways leading to hyperuricemia are summarized in Figure 1.

Hyperuricemia has also been linked to histological features of atherogenesis, such as increased thickness of carotid intima-media, and in clinical studies to symptomatic atherosclerotic plaques and plaque rupture [11,12,13,14], suggesting that uric acid may play a key role in the atherosclerosis progression and plaque rupture. This was confirmed by a recent study conducted with optical coherence tomography (OCT) performed during percutaneous coronary intervention (PCI) in 346 with acute coronary syndrome. The uric acid-HDL-C ratio, a worthy inflammatory and metabolic biomarker, correlated with diameter stenosis and area stenosis. Moreover, the UHR was found to be independently associated with plaque rupture, erosion, and thrombus [15]. Based on this evidence, several studies attempted to evaluate the existence of a pathophysiological connection between uric acid metabolism and brain health, potentially leading to cognitive dysfunction and dementia. The evidence on this relationship is somehow conflicting with some studies supporting the hypothesis that elevated uric acid levels could reduce a patient’s risk of Alzheimer’s disease, slow down the decline of cognition, and delay the progression of Alzheimer’s disease, while other evidence achieves the opposite positions [16,17,18]. Observational studies also indicate a U-shaped association between uric acid and brain diseases, Mendelian randomization studies do not support this as a causal association [19].

These discrepancies appear even more interesting when observed from an evolutionary perspective. During the mid-Miocene, mutations involving the promoter region of the uricase gene occurred and led to a stepwise loss of this enzymatic activity [20]. The increase in loss of function mutations in the uricase gene rendered hominoids (apes and humans) to have higher urate concentrations compared to other mammals, also because of extensive reabsorption of uric acid in the kidney. Humans’ relatively high uric acid levels have raised questions about its evolutionary advantages during hominid evolution [21,22]. Indeed, evolutionary physiology has treated uric acid as a molecule essential to human health, not a harmful waste. Due to the potent antioxidant activity of uric acid, the evolutionary benefit could have been due to protective effects against several neurodegenerative diseases. Therefore, it has been speculated that it could have interesting effects on neuronal development and function [22]. In today’s societies, people consume significantly more fructose and purine-rich foods, such as meat, which both generate uric acid. On the other hand, due to the loss of uricase, humans not only have higher uric acid levels than most other mammals, but they are also no longer able to regulate these levels effectively [23,24]. Consequently, circulating uric acid levels in the general population are continuously increasing. Nowadays, they are relatively higher compared to those observables in subjects whose life conditions are similar to those of our prehistoric ancestors. Thus, the hypothetical evolutionary advantages derived from uricase gene silencing, which have caused a moderate increase in serum uric acid levels, might have now turned into detrimental effects also on brain functioning [20]. In other words, uric acid seems to be characterized by a biological ambivalence mainly depending on the concentrations achieved in biological fluids, the chemical microenvironment [24,25,26], and the different degrees of involvement of the diverse pathophysiological mechanisms underlying uric acid-related vascular damage [27,28,29], thus potentially influencing brain health in opposite directions. In this regard, it is worth mentioning that uric acid concentrations are different in plasma and cerebrospinal fluid (CSF). A cutoff of 360 mol/L (6 mg/dL) for serum uric acid levels is generally used to define hyperuricemia, as the risk of clinical gout seems to appear around this value [24,30]. In addition, also the relationship between uric acid metabolism and cardio-nephro-metabolic disorders seems to have a steeper increase starting from this threshold [24]. Uric acid is commonly detectable in CSF [31,32]. Although the brain seems to have the capacity to generate uric acid, it is likely that the concentrations of uric acid achieved in CSF fluid mainly depend on plasma uric acid concentration and blood–brain barrier integrity [31]. Indeed, a positive correlation between concentrations of UA in plasma and CSF has been demonstrated in patients with moderate AD [31]. In these subjects, UA concentrations in CSF are 10-fold lower in comparison to those observed in plasma and are directly correlated with the CSF Albumin Index, a marker of brain–blood barrier integrity [31].

Hence, due to the lack of definite information on the direction of the relationship between uric acid and cognitive function, it could be reasonable to discuss the potential pathophysiological mechanisms by which uric acid metabolism might influence brain health and cognitive functioning. Furthermore, understanding these mechanisms could have therapeutic implications. In this narrative review, we attempt to elucidate the influential role of uric acid metabolism in cognitive functioning by discussing pathophysiological mechanisms putatively involved, being well aware that none of them can be considered one-sided due to the complexity of the human organism.

## 2. Monosodium Urate Deposition and Cerebrovascular Damage

Epidemiological observations demonstrate a significant increase in the risk of atherosclerotic disease in patients with gout [33,34]. A recent large-scale study demonstrated that patients diagnosed with gout exhibit a 58% elevated risk of developing cardiovascular disease. Above all, in younger patients and women with gout, the relative risk is 88% higher in the latter group when compared with women without gout. Furthermore, the study indicated that this elevated risk is applicable to a wide range of cardiovascular disorders, including heart failure, ischemic heart disease, arrhythmias, and stroke [35]. Several cohort studies outline an increased risk of stroke in patients with gout [19]. In a UK dataset, the rate ratio for stroke following hospital admission for gout was increased by 75%, covering all types of strokes (ischemic, hemorrhagic, unspecified) [36]. Similarly, in a cohort of patients with incident gout over 50 years of age, an increased risk for cerebrovascular disease (including cerebrovascular attack and transient ischemic attack) was observed in women [37]. In contrast, men with incident gout had an increased risk of transient ischemic attack but not of cerebrovascular attack [37]. In a US claims database, gout posed the same risk for incident stroke as diabetes mellitus [38]. Notably, a Taiwanese health insurance database showed that patients with gout treated with urate-lowering therapy had an almost 50% lower risk of incident stroke compared to those not treated with urate-lowering therapy, suggesting that urate-lowering therapy potential cardiovascular protective effects might extend to cerebrovascular disease [39]. More recently, an increased likelihood of stroke, as well as ischemic heart disease and heart failure, have been observed in individuals with gout [40]. Although, a recent Mendelian randomization analysis yielded no compelling evidence to suggest a causal association between genetically elevated serum urate levels and an increased risk of coronary artery disease or stroke. The findings of the study might challenge the hypothesis that uric acid plays a direct causal role in the development of cardiovascular disease [41]. However, behind the same UA levels, we must imagine different phenotypes, identifiable according to prevalent pathophysiological mechanisms individually involved in the relation between UA metabolism and cardiovascular disease, with a probable pivotal role for uric acid generating pathway rather than uric acid per se [29].

However, changes in brain cortical thickness have been described in gouty in patients with thicker cortices in the left postcentral, left supramarginal, right medial temporal, and right medial orbitofrontal regions and thinner cortices in the left insula, left superior frontal, right pericalcarine, and right precentral regions [42]. These alterations could be attributed to the combined effect of disease damage and physiological compensation. More recently, a prospective cohort study found smaller global and regional brain volumes in subjects with a history of gout [43]. These data suggest that gout may be causally related to brain structure changes, increasing the vulnerability to dementia.

**Figure 2:**
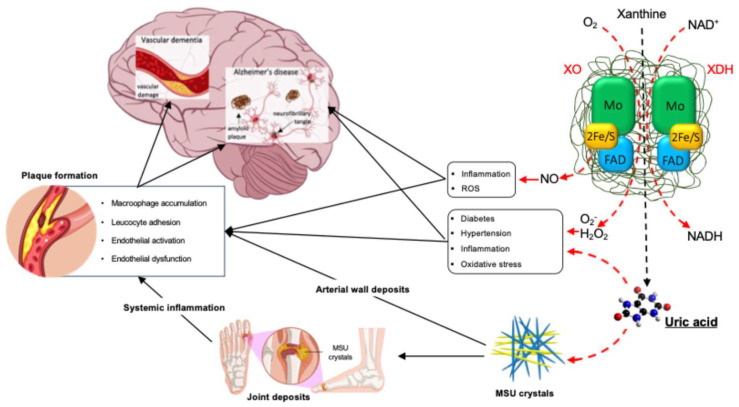
Mechanism potentially involved in the pathophysiology of cognitive dysfunction related to uric acid metabolism. Local and systemic inflammation and oxidative stress, induced by xanthine oxidase activity and monosodium urate deposition in joints and arteries, represent an important indirect mechanism linking uric acid metabolism, serum uric acid abundance, and cognitive dysfunction, both of vascular and non-vascular origin. Dash and red arrows represent a direct product of uric acid and its production process. Continuous black arrows indicate the secondary effect of uric acid and its production. ROS: Reactive Oxygen Species; MSU: Monosodium Urate; H_2_O_2_: Hydrogen Peroxide; O_2_^−^: Superoxide Anion; XO: Xanthine Oxidase; XDH: Xanthine Dehydrogenase; FAD: Flavin Adenine Dinucleotide; NO: Nitric Oxide: NADH: Nicotinamide Adenine Dinucleotide.

From a pathophysiological perspective, the association between gout and cerebrovascular damage could be explained by the systemic inflammation related to the deposition of monosodium urate crystals within the joints, characteristic of both acute and chronic gout [44,45]. Indeed, chronic vascular inflammation represents the main pathophysiological mechanism underlying the onset and progression of atherosclerotic disease [46,47,48,49]. Monosodium urate crystal deposition is a hallmark of gout [50]. Under high uric acid concentration conditions, appropriate temperatures, and pH values, monosodium urate crystals form and deposit in local tissues, triggering acute and chronic inflammatory responses [50,51]. In gout patients, monosodium urate crystal deposition can persist for a long time, even until the advanced stages [50,51]. Systemic inflammation has been observed in gout remission, with monosodium urate crystal deposition occurring at intercritical and advanced stages, characterized by activated inflammatory pathways, enhanced inflammatory cell interactions, and increased arachidonic acid metabolic activity [51]. In this regard, Andrés et al. [52] reported a significant increase in coronary calcification and monosodium urate deposits in knees and metatarsophalangeal joints in patients with asymptomatic hyperuricemia. Even more interesting, Klauser et al. [53] demonstrated by dual-energy computed tomography cardiovascular monosodium urate deposits, as confirmed by polarized light microscopy. Cardiovascular monosodium urate deposits were detected by dual-energy computed tomography significantly more often in patients with gout than in controls and were associated with higher coronary calcium scores. Although association does not prove causation, monosodium urate deposits within the vessel wall might likely play a relevant role in the pathophysiology of atherosclerotic damage, thus contributing to the onset and progression of cognitive dysfunction up to vascular dementia and Alzheimer’s disease [4] (Figure 2). This point appears quite relevant since the evidence of monosodium urate crystal deposition represents an indication for urate-lowering treatment aiming to promote the dissolution of monosodium urate deposits and prevent the formation of new accumulations. This approach also has the biological possibility to exert cardiovascular and brain protection by reducing both vascular and systemic inflammation.

Both MCU and soluble uric acid might directly activate inflammasome NPSL3. Both locally deposited uric crystal and circulating soluble uric acid may contribute to both local and systemic low-grade inflammation, characterizing cardiovascular and cerebrovascular disease [54].

## 3. Direct Influence of Uric Acid on Vascular and Brain Health

The vascular inflammation promoted by the deposition of monosodium urate crystals cannot entirely explain the relationship between uric acid and cognitive dysfunction, with this relationship also reported in subjects with uric acid levels in the normal to high range, largely below the precipitation threshold of MSU that occurs at 6.4 mg/dL (aqueous solution, 37 °C, pH 7.4) [55,56]. Thus, if a negative influence of uric acid on brain health exists, it should be at least in part independent of the precipitation of monosodium urate crystals. This aspect is relevant since several lines of thinking suggest a favorable effect of uric acid on brain functioning.

The ancient aphorism of the English physician Thomas Sydenham, “gout kills more wise men than simple”, was based on the evidence of greater intelligence and creativity of some gouty patients. In the 1950s, Orowan [57] proposed that elevated uric acid levels might have benefited early hominoids due to their potential neurostimulant properties, given their chemical similarity to caffeine. Several studies have identified relationships between uric acid levels and IQ testing [58], achievement-oriented behavior [59], and school performance [60], although these associations are generally weak. Additionally, research has shown that uric acid can increase locomotor activity in rats [61], rises with emotional or physical stress [62], and may be linked to hyperactivity in children [63]. The aforementioned studies suggest the intriguing possibility that uric acid may favorably influence cognitive function. In this regard, in an elegant study by Patil et al. [64], higher serum uric acid levels were found in medical students categorized under a genius and superior high IQ compared to those with normal or borderline IQ, with evidence of a highly significant positive correlation between serum. Besides the possibility that serum uric acid and IQ have the contribution of partly common gene loci to their two traits, it is worth mentioning that the large majority of subjects with above-normal IQ have circulating levels of uric acid in the high–normal range, i.e., below 6 mg/dL [24]. This aspect is particularly relevant in the data interpretation since it has been well established that uric acid can modify its biological properties based on the concentrations reached in the biological fluids and concomitant environmental conditions [25], thus potentially manifesting a biological ambivalence [28,65]. In fact, uric acid accounts for approximately 60% of antioxidant capacity in the plasma by scavenging ROS and chelating metal ions [66]. Conversely, uric acid has been shown to exhibit pro-oxidant effects, including its ability to impair nitric oxide (NO) production and decrease its bioavailability leading to endothelial dysfunction [19,33,67]. Indeed, the antioxidant properties described for low serum uric acid levels can turn into pro-oxidant properties for high–normal serum uric acid levels [23,28].

In this regard, experimental models demonstrated that uric acid may negatively impact the pathophysiology of Alzheimer’s disease by promoting neuritic/cytoskeletal lesions and reduction of neuronal cell viability [68] to a degree similar to the neurotoxic effects induced by fibrillary amyloid β 25–35 [69]. The effects of uric acid on cell viability are evident starting at 40 µM, with lower concentrations not significantly influencing cell biology. Interestingly, the reduction in cell viability due to uric acid exposure has been observed at a dose achievable in CSF under conditions of mild hyperuricemia (i.e., 400 µM) [24]. Indeed, it has been shown that in individuals with mild cognitive impairment, the uric acid concentration in CSF is determined by its plasma concentration [31]. Each μmol/L increase in plasma uric acid was associated with a 5% increase in CSF uric acid [31]. This positive correlation and the tenfold higher plasma uric acid level compared to CSF in these subjects suggest that uric acid is produced peripherally, with its access to the brain limited by the blood–brain barrier. In this regard, specific uric acid transporters have been described in ependymal cells [70]. These data suggest a dose-dependent effect of uric acid on neuronal cell survival. Moreover, uric acid enhanced the neurotoxic effects of hydrogen peroxide even at the lowest tested concentration (20 µM), although uric acid alone did not affect cell viability at this dose. This second finding suggests that concomitant factors influence the final effect of uric acid on neuronal cell biology. According to this data interpretation, uric acid can amplify cell viability reduction induced by oligomeric amyloid β 1–42 and fibrillary amyloid β 25–35 in neuronal cells in culture. These conditions could simulate the early phase [71] and the late stage [72,73] of Alzheimer’s disease pathophysiology, respectively. Additionally, uric acid exacerbated the harmful effects of amyloid β on neuronal cell connections by promoting synaptic disconnection of dystrophic neurites [28].

Based on this evidence, it is intriguing to speculate that in the presence of hyperuricemia, the diffusion of uric acid through the blood–brain barrier could increase its concentrations in CSF to levels that might harm cell biology by promoting the onset and/or progression of neuronal damage. The impaired blood–brain barrier, which can occur in patients with Alzheimer’s disease [31], cerebrovascular disease [74], or high blood pressure [75,76], could further contribute to reaching potentially toxic levels of uric acid in the CSF. Indeed, the compromised integrity of the blood–brain barrier is associated with increased CSF uric acid levels [31]. Although in vitro evidence suggests that uric acid could exert neurotoxic effects and can accelerate neuronal damage related to amyloid β both in the early and advanced phase of Alzheimer’s disease [68], data cannot be directly translated in vivo. In fact, in contrast to the in vitro evidence, recent meta-analyses, which included a large number of patients, were conducted and showed that patients with gout or hyperuricemia had a lower risk of Alzheimer’s disease [77,78]. In fact, despite the evidence on the potential enhancing role of uric acid in the Aβ neurotoxic effect, experimental data on the direct interplay between uric acid and Aβ deposition are conflicting. Xiao Q. and colleagues demonstrated that uric acid activates transcriptions factor EB (TFEB)-related signaling pathways, promoting a cascade leading to microglia autophagy and Aβ degradation, thereby improving cognitive function in AD model mice and suggesting an inverse relationship between uric acid levels and Aβ deposition [79]. On the other hand, studies proved that the integral membrane protein 2B (ITM2B) not only inhibits amyloid precursor proteolysis, reducing the Aβ production [80], but also reduces kidney urate reabsorption, inhibiting glucose transporter 9 (GLUT9) activity, partially explaining mechanisms underlying a direct relationship between serum uric acid levels and Aβ deposition [81].

Besides this potential influence of increased uric acid concentration on neuronal cell biology, the involvement of uric acid-related cerebrovascular dysfunction should also be considered. Indeed, some evidence describes direct vascular damage caused by uric acid because of oxidative stress leading to endothelial activation and dysfunction [20,33]. In this regard, Patetsios et al. [82] performed a qualitative and quantitative measure of uric acid in atherosclerotic plaques from carotid endarterectomy specimens. The study showed that increased uric acid levels were present along with cholesterol only in plaques of atherosclerotic patients but not in nonatherosclerotic control specimens. Hence, this study further supports the pathophysiologic role of uric acid in atherosclerosis. Even more interesting, Nardi et al. [83] recently demonstrated the expression of uric acid in carotid atherosclerotic plaques, with higher expression in carotid plaque specimens from symptomatic (including stroke, transient ischemic attack, amaurosis fugax) versus asymptomatic patients. Uric acid expression in carotid plaques was positively correlated with serum uric acid levels and associated with inflammatory markers expressed in carotid plaques. Serum uric acid levels were significantly higher in symptomatic than in asymptomatic patients and are independent predictors of major adverse cardiovascular events and all-cause death [83]. These data suggest that in patients with carotid atherosclerosis, the presence of uric acid in the carotid atheroma may play a key role in inflammation, which may determine plaque vulnerability and subsequent rupture, thus providing a mechanistic explanation for ischemic cerebrovascular damage. Notably, it has been recently demonstrated that serum uric acid levels are associated with the echogenic features of carotid plaque vulnerability [84], which, in turn, is associated with worse cognitive performance and more rapid progression of cognitive dysfunction in elderly subjects with atherosclerotic disease [85].

Thus, we should consider the direct influential role of uric acid on brain tissue and vasculature directly related to increased exposure to this end product of purine metabolism, independent of the presence of monosodium urate crystal deposition (Figure 2). For patients with this asymptomatic hyperuricemia, an indication for a urate-lowering treatment does not exist yet, although some epidemiological evidence suggests possible cognitive benefits, mainly with xanthine oxidase inhibitors [86,87,88,89].

## 4. Influence of Xanthine Oxidase on Vascular and Brain Health

**Figure 3:**
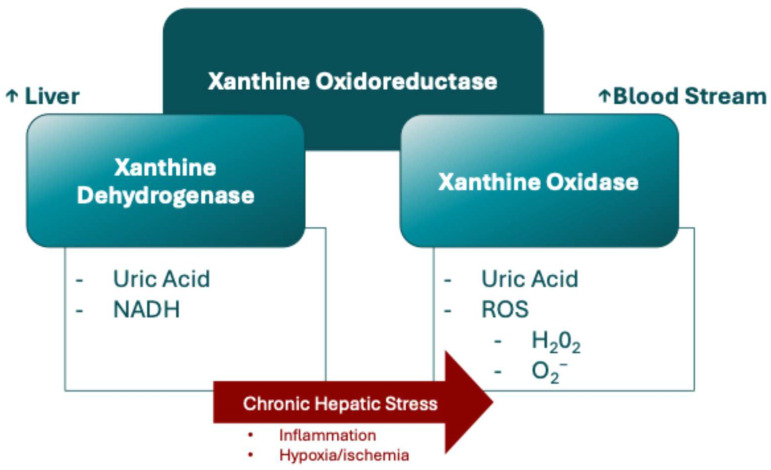
Main reaction and interaction involving xanthine oxidoreductase complex. Nicotinamide Adenine Dinucleotide. ROS: Reactive Oxygen Species; H_2_O_2_: Hydrogen Peroxide; O_2−_: Superoxide Anion; XO.

It has been recently proposed that xanthine oxidase mediates chronic stress-induced cerebrovascular dysfunction and cognitive impairment [90]. Xanthine oxidoreductase catalyzes the oxidation of hypoxanthine to xanthine and xanthine to uric acid, and it exists in two forms: xanthine dehydrogenase and xanthine oxidase [91,92]. While xanthine dehydrogenase uses NAD^+^ as an electron acceptor, xanthine oxidase transfers electrons to molecular oxygen, producing superoxide anions and hydrogen peroxide, and is most prevalent during proinflammatory conditions, including chronic stress [91,92]. Xanthine dehydrogenase is primarily transcribed and translated in the liver, exhibiting the highest specific activity there. However, during hepatic stress, such as inflammation, hypoxia, or ischemia, xanthine dehydrogenase can be released from hepatocytes into the bloodstream, where plasma proteases rapidly convert to xanthine oxidase (Figure 3) [91,93].

Xanthine oxidase has a high affinity for glycosaminoglycans on the vascular endothelium, and its immobilization on the endothelial surface induces endothelial dysfunction through oxidant production [84]. Xanthine oxidase generates reactive oxygen species, and when hyperactivated, it can contribute to oxidative stress, thereby enhancing cell injury caused by uric acid [27,28,90]. In this process, oxidative agents participate in the oxidation of macromolecules with subsequent activation of cell death signals and compromised structural integrity of plaques, possibly leading to cerebrovascular events [94,95].

Similarly, xanthine oxidase has been associated with proinflammatory cytokines such as IL interleukin-1α and interleukin-1β and reactive oxygen species generation in macrophages [96]. It is intriguing to consider that the same uric acid concentration in biological fluids could have different effects on cell biology, depending on the primary cause of the concentration, whether increased production by xanthine oxidase or impaired renal excretion [29]. The hyperactivity of xanthine oxidase increases uric acid generation and amplifies uric acid potential armful activity [27,29]. Interestingly, xanthine oxidase has been found in plaques from carotid endarterectomy specimens but not in nonatherosclerotic control specimens, supporting the pathophysiologic role of uric acid in atherosclerosis [82]. Animal studies have shown that pharmacological inhibitors of xanthine oxidase inhibited the progression of atherosclerosis, lipid accumulation, and calcification in ApoE^−/−^ mice [97,98]. Additionally, the expression of xanthine oxidase in macrophages is related to oxidative agents and the production of proinflammatory markers in atherosclerotic vessels [97].

Even more interesting, xanthine oxidase expression has been found to be higher in carotid atherosclerotic plaques from symptomatic versus asymptomatic patients [99]. In addition, macrophage markers have been found colocalized with regions of xanthine oxidase expression in the shoulder area of the carotid atherosclerotic plaque, and the higher percentage of macrophages expressing xanthine oxidase was associated with symptomatic plaque [99]. Serum uric acid levels were significantly higher in patients with symptomatic carotid plaque than asymptomatic carotid atherosclerotic plaques and directly associated with serum uric acid. Taken together, this evidence suggests that increased xanthine expressed in macrophages and serum uric acid levels may be a mechanism of plaque destabilization in patients with carotid artery disease.

A direct influence of xanthine oxidase activity on brain health should also be considered. A metabolomic study of cerebral spinal fluid found higher hypoxanthine and xanthine levels in subjects with mild cognitive impairment than in controls, and xanthine concentration was also higher in subjects with Alzheimer’s disease [100]. In these subjects, uric acid levels were also 25% higher in subjects with than in normal controls, and uric acid correlated with total tau protein when controls, mild cognitive impairment, and Alzheimer’s disease measurements were combined [100]. Interestingly, a recent study demonstrated a dose-dependent response to xanthine oxidase inhibitors regarding vascular brain damage [101], suggesting that this metabolic pathway plays a significant role in maintaining brain vessel integrity, which, in turn, affects uric acid concentration in CSF [31]. In this context, a recent experimental study demonstrated a role for xanthine oxidase in mediating chronic stress-induced cerebrovascular dysfunction [96]. In particular, mice who underwent eight weeks of unpredictable chronic mild stress exhibited increased liver xanthine oxidoreductase production and liver free radical formation, including increased hydrogen peroxide production, with increased circulating xanthine oxidase. Chronic stress also increases hydrogen peroxide production in brain tissue, specifically within the cerebral vessels. The administration of the xanthine oxidase inhibitor febuxostat prevented all these effects. Remarkably, febuxostat also prevented the onset of the memory deficits noted in mice who underwent chronic stress. These data suggest that xanthine oxidase plays an important role in cerebrovascular dysfunction related to chronic stress [90]. In addition, it was proven that febuxostat modulates 15 long noncoding RNAs related to maintaining the blood–brain barrier (BBB), suggesting a role of febuxostat on BBB integrity after intracerebral hemorrhage [102].

Thus, a third, and likely more relevant, pathophysiological mechanism theoretically involved in uric acid-related cognitive dysfunction is represented by xanthine oxidase overactivity due to genetic or acquired factors or to its overfeeding, for instance, because of an increased dietary intake of fructose or purine-rich foods (Figure 1). According to this interpretative hypothesis of current evidence, some studies found that uric acid-lowering therapy reduced the risk of dementia compared with that in subjects with untreated gout [86,87,88]. In one study, using the xanthine oxidase inhibitor febuxostat reduced the risk of dementia by 80% [88]. Another study reported a dose-dependent relationship, with higher doses of allopurinol and febuxostat providing greater protection [89]. Table 1 summarizes evidence on the association between uric acid, cerebrovascular damage, and cognitive function.

## 5. Conclusions

Uric acid has historically been viewed as a biologically inert waste product of purine metabolism, able to crystallize at high concentrations. Nonetheless, in recent years, growing scientific evidence has shown that uric acid may exert a wide array of relevant biological effects which could be dual, including pro- and antioxidant actions, probably depending on a very complex interplay of factors that include the concentration of uric acid, the nature and concentration of free radicals, the presence and concentration of other antioxidant molecules, and the various cascades involved. This biological ambivalence has now acquired clinical relevance because circulating uric acid levels in the general population are continuously increasing and are nowadays quite higher compared to those observed in subjects whose life conditions are similar to those of our prehistoric ancestors. Thus, the hypothetical evolutionary advantages derived from a moderate increase in serum uric acid levels might have now turned into detrimental effects on several apparatus, including the nervous system. Behind the same uric acid levels, we must imagine different phenotypes, identifiable according to prevalent pathophysiological mechanisms individually involved in the relation between uric acid metabolism and cognitive dysfunction. Indeed, the relationship between uric acid metabolism and cognition seems to be ambivalent, with some benefits for brain health deriving from “healthy” uric acid levels, which could turn in potentially dangerous effects depending on the chemical microenvironment, concentrations achieved in biological fluids as well as the prevalent pathophysiological mechanism responsible for the increase of uric acid, that is increased production, reduced excretion or both.

Although the pathophysiological journey described in this narrative review might seem too speculative, each step is supported by robust scientific evidence. The bi-directional biological effects of uric acid may have clinical implications. Firstly, increasing scientific evidence suggests that uric acid should be considered a potential determinant of brain health. Emerging data support re-evaluating the notion of “asymptomaticity” in chronic hyperuricemia and endorsing a threshold value of <6.0 mg/dL (<360 μmol/L) to identify truly “healthy subjects”, regardless of age and gender [24]. Secondly, due to the potential pathophysiological role of both systemic and vascular inflammation related to monosodium urate deposits, it is reasonable that significant brain protection could result from guideline-based urate-lowering treatment in patients with monosodium urate deposit disease. Finally, from a pathophysiological perspective, we should consider the phenotype “hyperuricemia” as quite heterogeneous since the various biological mechanisms underlying the influence of uric acid metabolism on brain health could operate differently in different patients. The different pathophysiological mechanisms could be partially imbricated but have different impacts on various subsets of patients. Therefore, the hypothetical use of urate-lowering treatment specifically for brain protection should always consider the predominant underlying pathophysiological mechanism in different individuals.

## Figures and Tables

**Table 1:** Clinical and experimental evidence on the association between uric acid, cerebrovascular damage, and cognitive function.

***Clinical Data*—Cerebrovascular Outcomes**			
**First Author** **(Year)**	**Study Design**	**Population**	**Results**
Seminog, O. (2013) [36]	Prospective data from UK registries	202,033 patients	The relative risk for all strokes was 1.71 (1.68, 1.75), ischemic stroke 1.68 (1.64, 1.73), hemorrhagic stroke 1.69 (1.61, 1.77), and stroke of an unspecified type 2.00 (1.95, 2.06).
Keenan, T. (2016) [41]	Mendelian randomization	82,091patients	Results were in contrast with previous prospective studies. Serum urate levels, increased by 1 standard deviation due to the genetic score, were not associated with type 2 diabetes mellitus, chronic heart disease, ischemic stroke, or heart failure.
Singh, J.A. (2017) [38]	Retrospective registry cohort study	1,338,501 patients	Compared with diabetes mellitus (DM) only, gout was associated with a similar risk of stroke (HR 1.02, 95% CI 0.95–1.10). Compared with patients with DM only, patients with both gout and DM had higher HRs for incident stroke (HR 1.42, 95% CI 1.29–1.56).
Yen, F.S. (2020) [39]	Retrospective database propensitive score matching cohort study	5218 patients	Incidence rates of hospitalized stroke were 0.6 and 1.0 per 100 person-years for urate-lowering therapy (ULT) users and nonusers, respectively, after adjusting for age, sex, residence, comorbidities, and medications. ULT users showed lower adjusted hazard ratios for hospitalized stroke (aHR: 0.52, *p* < 0.001). The effect of uricosuric agents on the decrease in hospitalized stroke risk indicated a dose–response relationship.
Kang, H.S. (2023) [40]	Retrospective database propensitive score matching cohort study	44,960 patients	In patients with gout, the incidences of stroke were slightly higher than those in controls (9.84 vs. 8.41 per 1000 person-years). After adjustment, the gout group had an 11% (95% confidence interval [CI] = 1.04–1.19) higher likelihood of experiencing stroke than the control group.
***Clinical Data*—Cognitive Outcomes**			
Schretlen, D.J. (2007) [55]	Cross-sectional data	96 patients	The multivariate-adjusted odds of poor verbal memory aOR 5.02 (1.24 to 20.30) and working memory aOR 4.25 (1.44 to 12.57) were higher in patients with mildly elevated (but normal) serum UA.
Ruggiero, C. 2009 [56]	Cross-sectional study	1016 patients	Demented persons had higher (uric acid) UA levels (*p* = 0.001), and the prevalence of persons affected by dementia increased across UA tertiles (*p* < 0.0001). Persons belonging to the highest UA tertile had a threefold (OR = 3.32; 95% CI: 1.06–10.42) higher probability of suffering from dementia syndrome.
Cicero, A.F.G. (2015) [18]	Cross-sectional data	288 patients	The only factors associated with the mini-mental state examination score were age (B = −0.058, 95% CI −0.108, −0.009, *p* = 0.022), LDL-C (B = −0.639, 95% CI −0.912, −0.411, *p* = 0.034) and SUA (B = −0.527, 95% CI −0.709, −0.344, *p* = 0.022).
MacIsaac, R.L. (2016) [101]	Prospective case–control study	4064 patients	Allopurinol use was associated with a significantly lower risk of stroke aHR 0.50, 0.32–0.80. In exposed patients, high-dose (≥300 mg) was associated with a significantly lower risk of stroke aHR 0.58, 0.36–0.94.
Engel, B. 2018 [86]	Cross-sectional study	137,640 patients	Patients with a hyperuricemia diagnosis had a slightly reduced risk for dementia (aOR 0.94, 0.89–0.98). The risk reduction was more pronounced for patients treated with anti-hyperuricemic drugs (aOR 0.89, 0.85–0.94, for regular treatment).
Sing, J.A. (2018) [89]	Database cohort study	1,710,000 patients	Gout was independently associated with a significantly higher hazard ratio of incident dementia, with an HR of 1.15 (95% CI, 1.12, 1.18); sensitivity analyses confirmed the main findings. Compared to age 65 to < 75 years, age 75 to <85 and ≥85 years were associated with 3.5 and 7.8-fold higher hazards of dementia.
Chuang, T.J. 2020 [87]	Retrospective case–control study	3242 patients	Benzbromarone decreased the risk of dementia (aOR, 0.81, 0.68–0.97), and its use for ≥180 days showed a significantly lower risk of dementia (aOR, 0.72, 0.58–0.89).
Min, K.H. (2021) [88]	Database cohort study	125,768 patients	Gout was independently associated with a significantly lower hazard ratio of incident dementia, aHR 0.63, 0.60–0.66. Moreover, febuxostat use significantly decreased incident dementia.
Pan, S.Y. (2021) [78]	Meta-analysis	2,155,959 patients	Gout and hyperuricemia did not increase the risk of dementia, with a pooled HR of 0.94 (95% CI 0.69 to 1.28), but might decrease the risk of Alzheimer’s disease (AD), with a pooled HR of 0.78 (95% CI 0.64 to 0.95).
Topiwala, A. (2023) [43]	Prospective data (UK Biobank)	303,149 patients	Gout is associated with a higher incidence of dementia (average over study HR = 1.60 [1.38–1.85]). The risk was time-varying, highest in the first 3 years after gout diagnosis (HR = 7.40 [4.95–11.07]) and then decreasing. Risks were higher for vascular dementia (average HR = 2.41 [1.93–3.02]) compared to all-cause dementia but not for Alzheimer’s disease (average HR = 1.62 [1.30–2.02]). Amongst asymptomatic individuals, in the linear model, there was an inverse association between urate and dementia incidence (HR = 0.85, 95% CI: 0.80–0.89).
Yao, Y. (2024) [77]	Meta-analysis	2,928,152 patients	Hyperuricemia (or gout) did not reduce the overall risk of dementia (OR/HR = 0.92, 95% CI: 0.81–1.05) and vascular dementia (OR/HR = 0.74, 95% CI: 0.53–1.05), but may have a protective effect against Alzheimer’s disease (OR/HR = 0.82, 95% CI: 0.70–0.96).
**In Vivo Study**			
**First Author** **(Year)**	**Study Design**	**Population**	**Results**
Bowman, G.L. (2010) [31]	Prospective study	32 patients	Cerebrospinal fluid (CSF) and plasma uric acid (UA) were positively correlated (*r* = 0.669, *p* = 0.001), and blood–brain barrier impairment was associated with higher CSF levels of UA (*p* = 0.028). Neither plasma nor CSF UA reached a significant association with rates of cognitive decline over 1 year.
Kaddurah-Daouk, R. (2013) [100]	Cross-sectional data	124 patients	At metabolic analyses, Alzheimer’s Disease (AD) subjects had elevated methionine (MET), 5-hydroxyindoleacetic acid (5-HIAA), vanillylmandelic acid, xanthosine, and glutathione versus controls. Mild cognitive impairment (MCI) subjects had elevated 5-HIAA, MET, hypoxanthine, and other metabolites versus controls. Metabolite ratios revealed changes within tryptophan, MET, and purine pathways. Initial pathway analyses identified steps in several pathways that appear altered in AD and MCI. A partial correlation network showed total tau most directly related to norepinephrine and purine pathways; amyloid-β (Ab42) was related directly to an unidentified metabolite and indirectly to 5-HIAA and MET.
Topiwala, A. (2023) [43]	Cross-sectional data (UK Biobank)	33,367 patients	From magnetic resonance imaging analyses, there were highly significant differences in regional grey matter volumes, particularly of mid- and hindbrain structures, such as cerebellum (beta = −9.91 × 10^−4^, *T* = −9.26, *p* = 2.25 × 10^−20^), pons (beta = −5.63 × 10^−4^, *T* = −6.23, *p* = 4.95 × 10^−10^) and midbrain (beta = −4.00 × 10^−4^, *T* = −5.15, *p* = 2.67 × 10^−07^) in gout and high urate. In addition, they had higher iron deposition (lower T2* and higher magnetic susceptibility) of several basal ganglia structures, including bilateral putamen (beta = 9.50 × 10^−4^, *T* = 9.25, *p* = 2.38 × 10^−20^) and caudate (7.62 × 10^−4^, *T* = 7.12, *p* = 1.13 × 10^−12^). Data were confirmed by genetic analyses for single-nucleotide polymorphisms associated with both elevated uric acid and gout.
Deng, F. (2024) [15]	Cross-sectional data	346 patients	The uric acid to HDL-C ratio (UHR) was found to be independently associated with plaque rupture, erosion, and thrombus. Furthermore, ROC analysis suggested that the UHR had a better predictive value than low-density lipoprotein cholesterol.
**In vitro and *Experimental* Studies**			
**First Author** **(Year)**	**Experimental Study Design**		**Results**
Santos, M.J. (2005) [69]	Primary Rat Hippocampal Neuron Cultures		Pretreatment with Wy-14.463, a peroxisome proliferator, prevents neuronal cell death and neuritic network loss induced by the Aβ peptide by increasing **both the number of peroxisomes and the catalase activity.**
Tomioka, N.H. (2013) [70]	Mouse Ependymal Cells		Urate transporter 1 (URAT1) was distributed throughout the ventricular walls of the lateral ventricle, dorsal third ventricle, ventral third ventricle, aqueduct, and fourth ventricle, but not in the non-ciliated tanycytes in the lower part of the ventral third ventricle. Antibody specificity was confirmed by the lack of immunostaining in brain tissue from URAT1 knockout mice.
Desideri, G. (2017) [68]	Human neuroblastoma Cells		The incubation of the cells model (SHSY5Y neuroblastoma cells incubated with amyloid β to reproduce an in vitro model of Alzheimer’s disease) with uric acid significantly reduced cell viability and potentiated the proapoptotic effect of amyloid β.
Burrage, E.N. (2023) [90]	Mouse		Blocking xanthine oxidase using febuxostat prevented the unpredictable chronic mild stress (UCMS)-induced impaired middle cerebral artery response, while free radical production and hydrogen peroxide levels were like controls in the liver and brain of UCMS mice treated with febuxostat. Further, UCMS + Feb mice did not have a significant reduction in working memory.
Zhang, C. (2023) [102]	Mouse		The authors found 15 long noncoding RNAs (lncRNAs) related to maintaining the blood–brain barrier (BBB). This study demonstrated that febuxostat protected the brain after intracerebral hemorrhage (ICH). Fifteen lncRNAs were regulated and were associated with the effects of febuxostat on BBB integrity after ICH.

## Data Availability

No new data were created or analyzed in this study. Data sharing is not applicable to this article.
